# Autonomic Imbalance in Cardiomyopathy and Heart Failure: From Neurobiology to Precision Neuromodulation

**DOI:** 10.1007/s11886-025-02288-7

**Published:** 2025-10-21

**Authors:** Aleksandr Talishinsky, Olujimi A. Ajijola, Sahib S. Khalsa

**Affiliations:** 1 UCLA Department of Medicine, Los Angeles, CA USA; 2 UCLA Division of Cardiology, Los Angeles, CA USA; 3 UCLA Semel Institute for Neuroscience and Behavior, Los Angeles, CA USA; 4 UCLA Department of Psychiatry, Los Angeles, CA USA

**Keywords:** Heart failure, Cardiomyopathy, Autonomic nervous system, Central autonomic network, Neuromodulation, Neurocardiology

## Abstract

**Purpose of the Review:**

This review provides a framework for understanding autonomic neural regulation of cardiac function and dysfunction, highlighting the anatomical and functional organization of the autonomic nervous system, from intrinsic cardiac neurons to central cortical control centers. We review pathways leading to autonomic dysregulation in heart failure (HF) and cardiomyopathy (CMY), and we discuss the potential for precision neuromodulation informed by biomarkers and neuroimaging.

**Recent Findings:**

We synthesize emerging insights into the molecular, inflammatory, and psychological mechanisms contributing to autonomic dysregulation in HF, and examine the clinical implications of impaired reflex arcs and persistent neurohormonal activation. Recent advances in neuromodulation, including vagus nerve stimulation, baroreflex activation therapy, spinal cord stimulation, cardiac sympathetic denervation and cortical neuromodulation demonstrate the potential to restore autonomic balance and improve HF outcomes.

**Summary:**

Autonomic imbalance, characterized by sympathetic overactivation and parasympathetic withdrawal, is a hallmark of HF and CMY, contributing to disease progression and adverse outcomes. While traditional pharmacotherapies target downstream neurohormonal pathways, neuromodulation offers the opportunity to intervene upstream, directly at pathophysiological nexus points. Ultimately, a shift toward personalized, circuit-specific neuromodulation strategies may offer new opportunities for treating autonomic dysregulation in HF and CMY.

## Introduction

Our aim in this review is to provide a mechanistic framework for understanding cardiac autonomic regulation in cardiomyopathy (CMY) and heart failure (HF). In order to accomplish this aim, we review recent findings documenting the role of autonomic dysfunction in CMY, and examine the therapeutic potential of neuromodulation for reversing autonomic dysfunction in CMY and HF. Autonomic imbalance, defined by sustained sympathetic activation and parasympathetic withdrawal, has long been recognized as a central feature of heart failure (HF). For example, a seminal paper by Kleiger et al. demonstrated a strong link between decreased heart rate variability, a marker of autonomic imbalance, and mortality after myocardial infarction [1]. Later studies confirmed this association [2, 3] and expanded it to nonischemic CMY [4, 5]. One notable experiment, the UK-Heart study, found that decreased HRV was associated with greater risk of death from progressive HF [6]. Since then, multiple reviews have highlighted a large body of evidence supporting the role of autonomic imbalance in the heart failure state [7–9]. More recently, novel contributors to autonomic dysfunction in HF have been identified, including glial cell hyperactivation, oxidative stress, and inflammation of the stellate ganglion [10–13], as well as psychological and environmental stressors [14–17]. Attempts at reversing autonomic neurohormonal imbalance in HF have demonstrated significant clinical benefit in the form of pharmacologic blockade of beta-adrenergic receptors or of the renin-angiotensin-aldosterone system, but these therapies are poorly tolerated in some patients. More recently, advances in neuroscience and neuromodulation have opened the door for testing and FDA approval of neuromodulatory therapies that hold promise in targeting autonomic contributors to HF [18, 19]. By integrating past and current findings on autonomic dysfunction in HF, this review emphasizes the therapeutic implications of emerging neuromodulatory approaches aimed at reversing autonomic imbalance across these pernicious cardiovascular conditions.

## Anatomy and Organization of Cardiac Autonomic Control

**Fig. 1:**
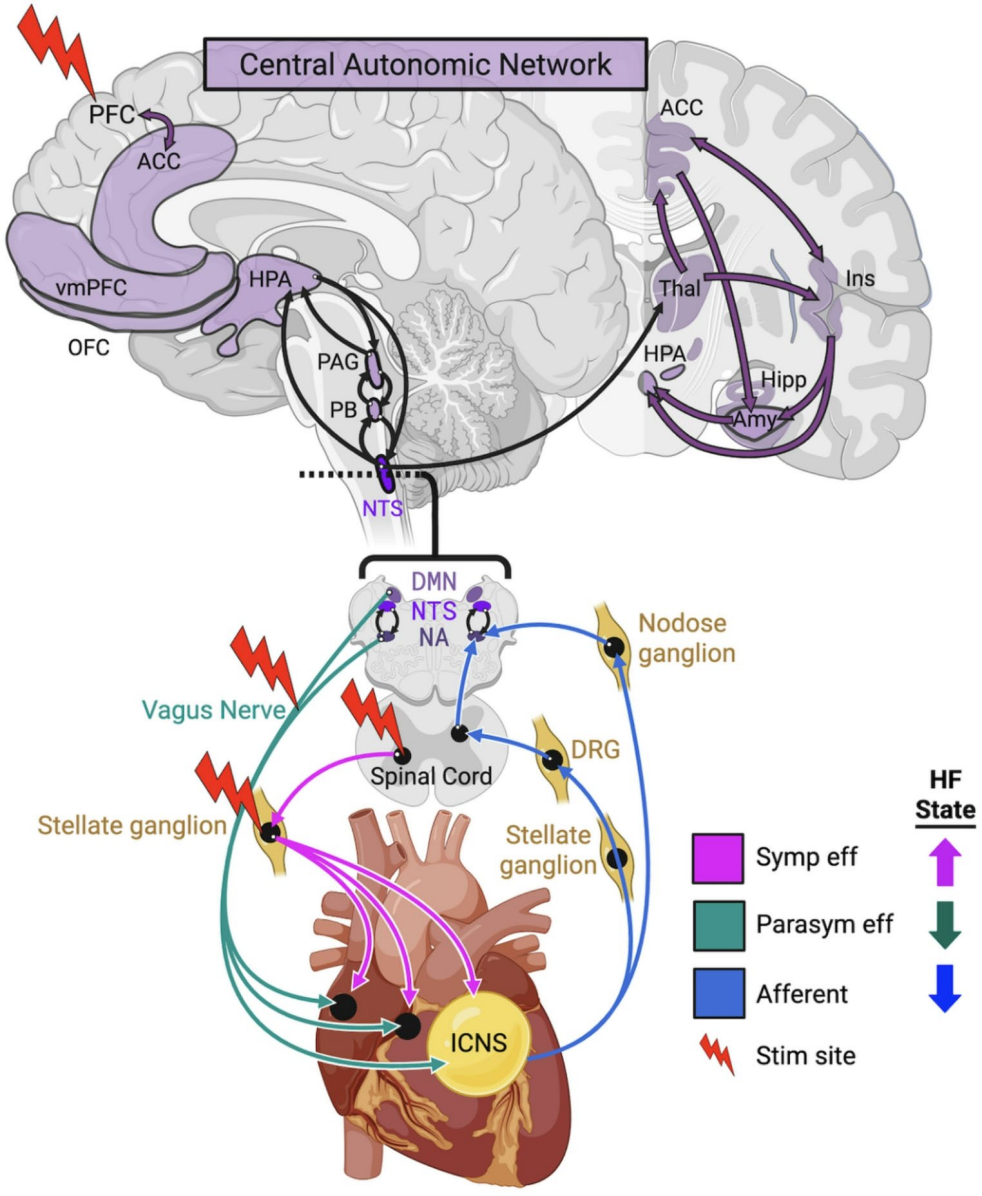
Neural Circuits of Cardiac Autonomic Regulation in Heart Failure. This figure illustrates autonomic innervation pathways connecting the heart’s intrinsic cardiac nervous system (ICNS) to peripheral nerve ganglia (nodose ganglion, dorsal root ganglion [DRG]), spinal cord, brainstem (including the nucleus ambiguus [NA], nucleus tractus solitarius [NTS], parabrachial nucleus [PB], and dorsal motor nucleus [DMN]), and higher brain regions within the central autonomic network (*shaded purple*), with efferent pathways projecting through the spinal cord and stellate ganglion to the cardiac sinoatrial and atrioventricular nodes. Note that afferents from the ICNS travel through the paravertebral (including stellate) sympathetic chains in a non-synaptic manner, synapsing in the DRG and then ascending via the spinal cord to brainstem targets. Neural pathways are color-coded: sympathetic efferents (*pink*), parasympathetic efferents (*cyan*), and autonomic afferents (*blue*). Central nervous system connections are represented by *black arrows* and *purple arrows*. *Red lightning bolts* indicate anatomical sites targeted for neuromodulation in heart failure (HF). The inset legend illustrates physiological alterations characteristic of HF, showing increased sympathetic and afferent activity and decreased parasympathetic activity. Note: the PB is also a major nexus point of autonomic and sensory pathways, with afferent and efferent PB-hypothalamus connections (not shown). Abbreviations: ACC, anterior cingulate cortex; Amy, amygdala; DMN, dorsal motor nucleus; DRG, dorsal root ganglion; Hipp, hippocampus; HPA, hypothalamus and pituitary; ICNS, intrinsic cardiac nervous system; Ins, insular cortex; NA, nucleus ambiguus; NTS, nucleus tractus solitarius; OFC, orbitofrontal cortex; PAG, periaqueductal grey; Parasym eff, parasympathetic efferent; PB, parabrachial nucleus; PFC, prefrontal cortex (including infra- and pre-limbic cortex); Symp eff, sympathetic efferent; Thal, thalamus; vmPFC, ventromedial prefrontal cortex. (Created in BioRender. Talishinsky, A. (2025) https://BioRender.com/atbt600.)

Autonomic regulation of the heart is achieved through a complex interplay of sympathetic and parasympathetic neural signals that span the entire neuraxis, from the peripheral to central nervous system (Fig. 1)(for detailed overviews, see [19–22]).

The anatomy of cardiac autonomic innervation can be subdivided into sympathetic and parasympathetic pathways, with the balance between these inputs regulating cardiac chronotropy, dromotropy, inotropy, lusitropy^1^, and metabolic function [23, 24]. Sympathetic fibers originate from the intermediolateral cell column of the thoracic spinal cord (T1–T5) and synapse in paravertebral ganglia, particularly the stellate ganglia, before projecting to the heart [25, 26]. In contrast, parasympathetic innervation of the heart is primarily mediated by the vagus nerve, originating in the medullary nuclei (nucleus ambiguus and dorsal motor nucleus) and exerting inhibitory control through acetylcholine at muscarinic receptors [27–29]. Sympathetic stimulation increases heart rate and contractility via β-adrenergic receptor activation, while vagal input slows heart rate and facilitates myocardial relaxation through acetylcholine-mediated signaling.

The functional organization of cardiovascular control occurs via hierarchical bidirectional signaling across numerous levels of the neuraxis [30, 31]. At the level of the heart, the intrinsic cardiac nervous system (ICNS)—the so-called “little brain on the heart”— incorporates afferent mechanosensory and chemosensory signals from neurites at cardiac and local vascular tissue [21]. The ICNS also receives efferent sympathetic and parasympathetic signals from the extracardiac intrathoracic ganglia and vagus nerve, respectively, and sends efferent sympathetic and parasympathetic projections to beta adrenergic and muscarinic acetylcholinergic receptors in myocardial tissue. As a nexus of afferent and efferent sympathetic and parasympathetic activity, the ICNS acts as both a crucial relay station and an independent modulator of autonomic function [32, 33].

Extra-cardiac neural circuits integrate afferent and efferent inputs to coordinate autonomic activity from the brain, spine, and autonomic ganglia. The stellate ganglion–heart–stellate ganglion loop supports localized feedback within the sympathetic chain; the spinal cord–heart–spinal cord loop via the dorsal root ganglia (DRG) integrates somatic and visceral sensory input and coordinates sympathetic output; and the brainstem–heart–brainstem loop transmits both sympathetic and parasympathetic signaling and incorporates feedback via vagal and ascending spinal afferents [32, 34]. Autonomic sensory inputs from other centers, including the kidney, skeletal muscle, and stretch- and chemo-receptors at the carotid body and aortic arch are incorporated at the level of the spinal cord and medulla to further modulate autonomic outputs to the heart. Of particular importance is the baroreflex, a cornerstone of hemodynamic homeostasis, which is significantly blunted in chronic heart failure [35, 36]. Baroreceptors in the carotid sinus and aortic arch mediate hemodynamic homeostasis via ascending signals to the brainstem nucleus tractus solitarius (NTS), which then sends efferent reflexive sympathetic and parasympathetic signals to maintain stable blood pressure and heart rate [37]. Similarly, the Bezold-Jarisch reflex modulates sympathovagal balance in response to various pharmacologic or mechanical stimuli detected in the heart’s ventricles [38]. The precise molecular underpinnings of the Bezold-Jarisch reflex were unknown until recently, when Lovelace et al. used single cell RNA sequencing, HYBRiD tissue clearing, and optogenetics to demonstrate a causal role of vagal afferent neuropeptide Y receptor Y2 signaling [39]. This study laid the groundwork for future efforts to dissect the genetic, anatomic, and functional underpinnings of autonomic reflexes, which may lead to novel targets for treatment of autonomic dysfunction in HF.

Above the brainstem, cortical centers including the insular cortex and anterior cingulate cortex modulate autonomic tone and cardiac output in response to both interoceptive and cognitive-emotional states [31, 40]. These cortical centers are part of the central autonomic network (CAN), a distributed neural system responsible for bidirectional regulation of autonomic outflow and integration of visceral feedback [40–43]. Subcortical and brainstem integrative hubs, including the thalamus, hypothalamus, NTS and parabrachial complex, are essential relays supporting the sensory processing of afferent cardiac signals [41, 44]. The importance of the brain and cervical spine in parasympathetic control are highlighted in heart transplant recipients, who undergo transection of the cervical vagus nerve, leading to significantly higher resting heart rates due to withdrawal of central parasympathetic tone [45, 46]. Vagal afferent signals from the ICNS and cardiac sensory neurites modulate CAN activity and in turn receive efferent output [47], thereby completing a bidirectional feedback loop that functions in parallel with all of the circuits mentioned above.

### Functional Roles of Autonomic Input in Cardiac Physiology

Sympathetic nervous system stimulation enhances cardiac chronotropy, inotropy, dromotropy, and lusitropy through β-adrenergic signaling by increasing intracellular calcium available for myocardial work [24]. In contrast, parasympathetic nervous system input via muscarinic receptors suppresses these processes, enabling myocardial recovery and energy conservation. In the resting state, sympathetic and parasympathetic efferent signals strike an intricate balance to modulate cardiac output.

Autonomic control of the heart influences not only rhythm and contractility but also tissue perfusion, energetic balance, and reparative processes. Beyond electrophysiologic modulation, autonomic inputs additionally influence local immune responses, angiogenesis, and intracellular regenerative pathways. Parasympathetic stimulation may even enhance reparative cytokine profiles and suppress pro-inflammatory signaling, although these effects remain incompletely characterized [48–50]. On the other hand, stress-related stimuli can activate the ANS through both top-down cortical input and bottom-up interoceptive signaling from the myocardium [51, 52]. The result is neuroendocrine activation that, when unchecked, contributes to maladaptive cardiac remodeling.

## Autonomic Dysregulation Leading to Heart Failure and Cardiomyopathy

In HF, chronic sympathetic activation and vagal withdrawal constitute a state of maladaptive neurohumoral imbalance. Circulating catecholamine levels are elevated in both heart failure with reduced ejection fraction (HFrEF) and heart failure with preserved ejection fraction (HFpEF), resulting in increased sympathetic drive and correlating with disease severity and prognosis [53–55]. This sympathovagal imbalance initially serves a compensatory role for reduced cardiac output, but over time becomes persistent through multiple mechanisms including cardiac beta-2 receptor desensitization [56, 57], cardiomyocyte hypertrophy [58], cardiac transcriptional reprogramming [59, 60], and neuroplastic changes in spinal and ganglionic circuits [61–63] (for reviews see [64, 65]). Together, these maladaptive mechanisms may be characterized as a dysfunction of allostatic regulation, in which cumulative external and internal stresses on the cardiovascular system result in maladaptive alterations of autonomic function [66]. Recurrent exposure to allostatic load, including risk factors such as high blood pressure, hyperlipidemia, hyperglycemia, inflammation, or metabolic syndrome, is associated with heart failure-related hospitalization and death [67]. This reflects a failure of the autonomic nervous system to adapt sustainably in the face of ongoing stressors. The ultimate result is a self-perpetuating positive feedback cycle of neurocardiac activation leading to a ‘downward spiral’ of HF and autonomic dysfunction.

Baroreflex desensitization is one well-characterized manifestation of autonomic dysregulation. The arterial baroreflex regulates blood pressure via rapid adjustments in heart rate and vascular tone. In HF, baroreflex sensitivity is reduced due to both central remodeling and afferent signaling alterations [35, 36]. Impaired baroreflex gain permits greater fluctuation in arterial pressure and contributes to episodic hypotension, diminished vagal tone, decreased heart rate variability, and persistent sympathetic overdrive.

Recent studies have highlighted novel cellular and molecular pathways contributing to autonomic dysfunction in HF. Inflammation of the stellate ganglion has been shown to potentiate chronic sympathetic overdrive in both animal and human clinical studies [10–12]. Local mechanisms including oxidative stress, cytokine activation, neurochemical remodeling, and neuronal nitric oxide synthase expression mediate increased norepinephrine release leading to sympathetic hyper-activation and decreased vagal tone in animal models [10, 68, 69]. Myocardial beta-1 receptor auto-antibodies have also been hypothesized to play a role in heart failure pathogenesis by increasing sympathetic tone [70, 71]. Serum from heart failure subjects is enriched for anti-beta-1 receptor auto-antibodies [72], whose presence predicts increased mortality [73], and depletion of these auto-antibodies has led to improved myocardial function in two small trials [74, 75]. By binding and stimulating the beta-1 adrenergic receptor, these auto-antibodies promote further autonomic imbalance and sympathetic hyperactivation. In turn, sympathetic hyperactivation has been shown to promote myocardial hypertrophy via the NLRP3 inflammasome and the extracellular ATP-P2 × 7 signaling pathway [76]. The NLRP3 inflammasome is thought to mediate myocardial damage in cardiomyopathy both indirectly via IL-1β release [77] and directly via pyroptosis [78]. By mediating myocardial hypertrophy in response to sympathetic tone, the NLRP3 inflammasome serves as an important molecular link between autonomic dysfunction and heart failure (for more detailed overviews, see [79, 80]). Vagal nerve stimulation has been hypothesized to improve sympathovagal balance in part by reducing these inflammatory pathways [49, 81].

Concomitant exogenous factors, such as psychological and environmental stressors [14, 15], are also gaining greater appreciation for their role in potentiating autonomic dysfunction in HF. Psychological stress and mood disorders such as depression have been associated with increased sympathetic tone and reduced vagal activity [82, 83]. In multiple large-scale cohort studies, depression was shown to carry an 18–21% increased risk of developing heart failure independent of other risk factors [84, 85] and up to 2-fold increased risk of cardiac events or death in subjects already diagnosed with HF [86]. The relationship between depression and HF is thought to be mediated at least in part by a common pathophysiologic pathway involving autonomic dysregulation [16, 17], but the underlying mechanisms are still unclear. Imaging studies have implicated the CAN in perpetuating autonomic imbalance in HF [87, 88], suggesting a possible brain substrate linking HF to cognitive-emotional dysregulation and mental health disorders. On a molecular level, adrenergic activation leading to the release of norepinephrine and neuropeptide Y from synaptic terminals in the myocardium is thought to mediate myocardial damage in stress cardiomyopathy [89]. These neuropeptides act via both direct myocardial toxicity [90] as well as indirectly via changes in myocardial perfusion [91]. Other neuropeptides, including calcitonin gene-related peptide, substance P, galanin, or vasoactive intestinal peptide, have also been implicated in adverse cardiac remodeling in heart failure [92]. The precise role of these neuropeptides in autonomic dysfunction is not yet clear, but they are co-released with autonomic neurotransmitters and have been suggested to play a role in the pathogenesis of multiple cardiomyopathies [93].

The pillars of contemporary heart failure treatment aim to restore autonomic neurohormonal balance. The most effective guidance-directed medical therapies to date—comprising β-blockers, angiotensin-converting enzyme inhibitors, angiotensin receptor-neprilysin inhibitors, and mineralocorticoid receptor antagonists—indirectly restore autonomic balance by suppressing contributory neurohormonal pathways [94, 95]. However, these guideline-based pharmacologic approaches do not target neural circuits directly and are often limited by intolerance or side effects, while direct modulation of autonomic circuits remains underexplored in clinical settings. In the next section we consider the role of neuromodulation strategies in management of autonomic dysregulation.

## Neuromodulation Strategies for Autonomic Rebalancing

Given the hierarchical organization of cardiac autonomic control, neuromodulation can be approached at multiple levels, from various cortical centers within the brain to peripheral autonomic ganglia (Table 1).

Higher-order cortical regions involved in stress and emotional processing are known to modulate autonomic tone. Direct and indirect modulation of higher cortical function has shown potential for reversing autonomic dysfunction in HF. Although indirect, interventions such as cognitive behavioral therapy, meditation, yoga, and biofeedback therapy influence higher cortical function by modulating cognition and emotions, and have been associated with reduced sympathetic output and improved functional capacity in HF populations in both observational and prospective randomized studies [96–100]. Early trials have shown potential for direct neuromodulatory interventions on higher cortical centers. In individuals with depression, transcranial magnetic stimulation (TMS) over the left dorsolateral prefrontal cortex was associated with decreased HRV compared to sham stimulation [101]. This effect was thought to be mediated by increased parasympathetic tone resulting from TMS [102–104]. However, TMS is limited by its shallow depth and broad field effect [105], which may constrain its clinical impact in HF. On the other hand, transcranial alternating current stimulation (tACS) can stimulate neural targets deeper within the brain but with less specificity [106]. Thus far, tACS of the prefrontal cortex has been reported to modulate sympathetic tone in cardiovascular and muscle tissue [107, 108], but it has not yet been trialed in deeper centers of autonomic regulation such as the anterior cingulate or insular cortices. Transcranial focused ultrasound (tFUS) represents another alternative capable of targeting focal, deep structures, but sometimes with lower precision due to beam deformation by skull shape/porosity [109, 110]; tFUS has also not been trialed for autonomic dysfunction or in patients with heart failure. TMS, tACS, and tFUS thus represent potential opportunities for therapeutic central nervous system modulation of autonomic and cardiac output, but await further study.

Vagus nerve stimulation (VNS) aims to restore parasympathetic tone through electrical stimulation of the cervical vagus nerve. Animal studies of VNS for HF have shown substantial promise, decreasing mortality by over 70% and improving LV function, neural remodeling, and glial hyperactivation in experimental models of HF [81, 111, 112]. Human clinical trials of implantable VNS have thus far yielded mixed results. The ANTHEM-HF trial reported improved ejection fraction and functional class [113], contrasting with neutral outcomes in the NECTAR-HF [114, 115] and INOVATE-HF [116] trials. This disparity may reflect differences in stimulation protocols, anatomical targeting, and patient demographics [117]. Compared to NECTAR-HF and INOVATE-HF, ANTHEM-HF subjects were younger and had less severe HF symptoms; ANTHEM-HF successfully delivered 10 Hz VNS at an average current of 2.0 mA with confirmed changes in HR dynamics, whereas NECTAR-HF used 20 Hz VNS with limited current delivery (average 1.4 mA) due to off-target effects, and INOVATE-HF delivered VNS at a much lower pulse frequency of 1–2 Hz due to the use of closed-loop stimulation aimed preferentially at peripheral nerve targets [117]. To synthesize these discrepant results under different stimulation parameters, Ardell and colleagues conducted experiments in canines to identify a “neural fulcrum” of effective VNS parameters with balanced afferent and efferent engagement [118]. Of the three studies listed above, only ANTHEM-HF effectively titrated stimulation frequency, amplitude, and pulse width to the “neural fulcrum”, defined as the operating point where a null or slightly bradycardic heart rate response is reproducibly evoked during the on-phase of VNS [19, 118]. This may explain in part the difference in trial outcomes. Alternative forms of vagal nerve stimulation, such as trans-auricular or trans-cutaneous vagal nerve stimulation, have not yet been tested in large-scale trials for heart failure. Future efforts at vagal nerve stimulation in humans may benefit from incorporating trans-auricular or trans-cutaneous stimulation, titrating stimulation more precisely to the “neural fulcrum” described above, or employing selective anatomically targeted stimulation of efferent vagal fibers for scalable heart rate modulation, which has recently been accomplished in animal models using micro-computed tomography fascicle tracing of the mid-cervical vagus nerve [119].

Baroreflex activation therapy (BAT) involves implantable electrical stimulation of the carotid sinus to engage baroreceptor afferents, thereby enhancing baroreflex sensitivity and suppressing central sympathetic outflow. The BeAT-HF trial prospectively tested BAT in 323 patients with severe HFrEF. Although the trial did not demonstrate a significant difference in its primary endpoint of cardiovascular morbidity and mortality, it demonstrated improved quality of life, reduced natriuretic peptide levels, and lower hospitalization rates with BAT compared to medical therapy alone [120, 121]. Larger randomized controlled trials may be necessary to better assess the full benefits of BAT with regard to cardiovascular morbidity and mortality.

Spinal cord stimulation (SCS) delivers electrical impulses directly to the thoracic spinal cord, with the goal of reducing sympathetic tone and increasing myocardial perfusion. Preclinical models suggest that SCS can stabilize cardiac electrophysiologic substrates and reduce arrhythmic risk [122, 123]. Early pilot studies in humans have shown improvements in cardiac functional capacity and angina burden [124, 125], although these were not reproduced in the larger DEFEAT-HF randomized controlled trial of SCS in 81 patients with HF [126]. As with VNS, further studies are needed to determine whether optimization of electrode placement and stimulation parameters produces maximal benefit of SCS in heart failure patients.

Stellate ganglion modulation via invasive or pharmacologic interventions has shown efficacy in suppressing ventricular arrhythmias. Bilateral surgical resection or chemical blockade of the stellate ganglion, with the goal of reducing efferent sympathetic arrhythmogenic input to the heart, has been used to prevent sudden death in high-risk patients with arrhythmias such as long QT syndrome or refractory ventricular tachycardia (VT) [127–131]. Based on these observational studies and a prior randomized controlled trial [132], there is currently a class IIb recommendation for cardiac sympathetic denervation in patients with refractory VT due to CMY [133]. More recent work has demonstrated the feasibility of transvenous stimulation of the cervical sympathetic chain, which may prove useful for either direct therapeutic applications or for targeting if used in combination with catheter ablative therapy [134]. Additional non-invasive techniques to target the stellate ganglion have included transcutaneous magnetic [135] or electrical [136] stimulation, both of which warrant further investigation due to their potential for broader implementation. A randomized trial testing different parameters of transcutaneous magnetic stimulation (theta burst versus low-frequency) for reducing VT burden is currently ongoing (NCT05599022). Theta burst stimulation provides bursts of high-frequency magnetic stimulation occurring approximately 5 times per second, thereby mimicking endogenous neuronal theta burst activity and improving induction of synaptic long-term potentiation [137, 138]. This trial may provide valuable results about optimal stimulation parameters for transcutaneous magnetic stimulation of the stellate ganglion to improve both VT burden and HF outcomes.

Finally, peripheral neuromodulatory therapies including renal denervation, Vein of Marshall ablation, and splanchnic nerve ablation may have potential for improving HF outcomes by reducing cardiac afterload, atrial fibrillation burden, and preload, respectively. Renal denervation has shown potential for the management of treatment-resistant hypertension [139], and has also been shown to reduce the burden of tachycardia and atrial fibrillation in the ERADICATE-AF randomized trial [140]. Through its effects in reducing hypertension and tachycardia, renal denervation has appeal as a HF treatment; it has demonstrated improved HF outcomes and reduced HF symptoms in small randomized studies [141–143], but there have been no large-scale, sham-controlled randomized studies of renal denervation in patients with HF to date. Similarly, Vein of Marshall ablation has shown a reduction in atrial fibrillation burden when added to catheter ablation alone in the VENUS randomized controlled trial [144], in which > 25% of subjects had comorbid HF. Ablation for atrial fibrillation, in turn, has been associated with significantly lower all-cause mortality and hospitalization rates in subjects with HF via the landmark CASTLE-AF trial [145]. However, Vein of Marshall ablation has not yet been tested specifically for patients with HF. In subjects with HFpEF, the REBALANCE HF trial for splanchnic nerve ablation demonstrated a favorable safety profile and a favorable reduction in pulmonary capillary wedge pressure and secondary HF outcomes in its lead-in phase [146], but it did not show significant reduction in primary or secondary outcomes for the 90 subjects in the randomized phase [147]. In summary, modulation of peripheral nerves in the renal veins, Vein of Marshall, and splanchnic veins directly target autonomic dysregulation in heart failure by reversing the downstream effects of sympathovagal imbalance; namely hypertension, tachycardia, and increased atrial fibrillation burden. These therapies have shown the potential for improving HF outcomes but are not yet proven in dedicated large-scale randomized trials.

## The Future of Cardiac Neuromodulation for HF

Neuromodulatory therapies for HF are quickly growing and have demonstrated potential for managing autonomic dysfunction in HF. Contemporary neuromodulatory therapies for HF have targeted anatomic foci of autonomic cardiac control outlined in the text above and in Table 1. As the era of personalized medicine approaches, we anticipate that current neuromodulatory approaches will evolve into individualized targeting strategies to ablate patient-specific aberrant foci driving neurohormonal imbalance. The practicing cardiologist may thus be tasked with selecting personalized neuromodulation strategies for each patient, based on biomarkers localizing foci of autonomic dysfunction, including different neurocircuit and molecular targets across the neurocardiac axis. Successfully accomplishing this approach will rely on adequate within-subject measurement of autonomic function, an ongoing area of research with promising recent developments in functional MRI [148–151], molecular [152–155], and electrophysiologic [156–158] biomarkers. Implementing neuromodulatory therapies for autonomic rebalancing may also address comorbid autonomic disorders in heart failure patients, such as hyperadrenergic postural orthostatic tachycardia syndrome [159], inappropriate sinus tachycardia [160], or dysautonomia [161]. Future work in biomarker development and optimization of neuromodulation techniques is likely to usher in an individualized approach, providing a potential alternative to current neurohormonal pharmacologic treatment strategies.

## Conclusions

Autonomic dysfunction in HF and CMY reflects a shift toward sympathetic dominance over parasympathetic activity. This imbalance is both a marker and driver of disease progression, contributing to arrhythmogenesis, impaired cardiac contractility, neuroinflammation, and maladaptive remodeling. Disruption of peripheral reflex loops and feedback between peripheral-central neurocircuitry contributes to persistent neurohormonal activation in HF. A growing body of evidence is beginning to support the feasibility of targeted neuromodulation across the heart-brain neuraxis. These interventions, directed at the level of the peripheral nerves, spinal cord, and cerebral cortex, may offer unique opportunities to restore autonomic balance and improve clinical outcomes.

**Table 1:** Neuromodulatory therapies for autonomic dysfunction in heart failure (HF)

Modality	Target	Evidence	Limitations	Relevance to HF
**Cognitive & Behavioral Therapies**	Higher cortical centers (indirect)	Observational and RCT data show reduced sympathetic output and improved functional capacity (e.g., Nolan 2005 [80], Freedland 2015 [83])	Indirect, non-specific effects	Supportive adjunct to reduce autonomic imbalance
**Transcranial Magnetic Stimulation (TMS)**	Left dorsolateral prefrontal cortex	Decreased HRV in depression patients (Udupa 2007 [85]); parasympathetic activation (Gulli 2013 [86])	Shallow penetration, broad field, not yet trialed in HF	Theoretical potential; not yet validated in HF
**Transcranial Alternating Current Stimulation (tACS)**	Prefrontal cortex (and possibly deeper brain structures)	Shown to modulate autonomic tone (Wong 2023 [92]); not yet tested in key autonomic centers (e.g., insula, cingulate)	Low specificity, limited HF application thus far	Promising, but untested in HF-specific autonomic regulation
**Transcranial Focused Ultrasound (tFUS)**	Deep brain structures	Theoretical capacity; not yet tested in HF or autonomic dysfunction	Beam distortion by skull, untested in HF	Early-stage, not yet explored in HF
**Vagal Nerve Stimulation (VNS)**	Cervical vagus nerve	Mixed results: ANTHEM-HF positive; NECTAR-HF, INOVATE-HF neutral. Animal studies showed mortality & LV function benefits	Trial disparities due to stimulation parameters and targeting	Clinically tested with mixed results; optimization needed
**Baroreflex Activation Therapy (BAT)**	Carotid sinus	BeAT-HF: improved QoL, NT-proBNP, hospitalization; no mortality benefit (Zile 2020 [104], Zile 2024 [105])	No primary endpoint success; further large trials needed	Validated improvements in secondary endpoints
**Spinal Cord Stimulation (SCS)**	Thoracic spinal cord	Preclinical and early pilot studies positive; DEFEAT-HF trial neutral (Zipes 2016 [110])	Limited human trial success; electrode placement/stimulation may need refinement	Potential benefit; parameter optimization required
**Stellate Ganglion Modulation**	Stellate ganglion	Surgical resection effective in arrhythmias; early non-invasive methods under study (Markman 2022 [119])	Invasiveness (surgery); non-invasive approaches need more evidence	Useful for arrhythmia suppression; potential for HF patients with high arrhythmic risk

## Data Availability

No datasets were generated or analysed during the current study.

## Footnotes

1 See Glossary for definitions.

## Glossary & Abbreviations

Chronotropy: rate of the heart’s systolic contractions (measured in beats per minute)
Dromotropy: velocity of electrical conduction through the heart and the atrioventricular node
Inotropy: force of myocardial contraction during systole
Lusitropy: rate of myocardial relaxation during diastole
Pyroptosis: An inflammatory form of programmed cell death triggering immune responses
BAT: Baroreflex activation therapy
CAN: Central autonomic network
CMY: Cardiomyopathy
DRG: Dorsal root ganglion
HF: Heart failure
HFrEF: Heart failure with reduced ejection fraction
HFpEF: Heart failure with preserved ejection fraction
ICNS: Intrinsic cardiac nervous system
NTS: Nuclear tractus solitarius
SCS: Spinal cord stimulation
tACS: Transcranial alternating current stimulation
tFUS: Transcranial focused ultrasound
TMS: Transcranial magnetic stimulation
VNS: Vagal nerve stimulation
VT: Ventricular tachycardia
